# Is the Health and Wellbeing of University Students Associated with their Academic Performance? Cross Sectional Findings from the United Kingdom

**DOI:** 10.3390/ijerph7020509

**Published:** 2010-02-11

**Authors:** Walid El Ansari, Christiane Stock

**Affiliations:** 1 Faculty of Sport, Health and Social Care, University of Gloucestershire, Gloucester, UK; 2 Unit for Health Promotion Research, Institute of Public Health, University of Southern Denmark, Niels Bohrs Vej 9-10, 6700 Esbjerg, Denmark; E-Mail: cstock@health.sdu.dk

**Keywords:** satisfaction, teaching and learning, student health, university, gender, educational achievement

## Abstract

This study explored the associations between health awareness, health behaviour, subjective health status, and satisfaction of students with their educational experience as independent variables and three outcomes of educational achievement as dependent variables. We undertook two simultaneous cross-sectional surveys among students from one University in the UK during 2008−2009. The first survey was a general health survey; the second survey measured students’ satisfaction with different aspects of their learning and teaching experience. Students’ registration numbers linked the responses of both questionnaires together, and subsequently linked the questionnaires to the university database to import the grades that students actually achieved in their studies. Generally, on average, students (N = 380) exhibited medium to high satisfaction with their educational experiences. In the multivariate regression analyses, students’ satisfaction with their educational experiences was not associated with any of the three indicators of educational achievement (actual module mark; perceived own performance; importance of achieving good grades). The associations of educational satisfaction, health, health behaviours, heath complaints and financial parameters with the three outcomes of educational achievement did not differ between male and female students. Each of the health, health behaviours, health complaints and financial parameters were selectively associated with only some but not all three indicators of student educational achievement. We conclude that the findings support a conceptual framework suggesting reciprocal relationships between health, health behaviour and educational achievement. Comprehensive health promotion programmes may have the potential to influence relevant predictors of educational achievement in university students.

## 1. Introduction

It has been argued that health is an important factor for academic achievement at school [1,2] and in higher education [3]. Consequently, in the context of universities or colleges, promoting the health and well-being of all members means promoting effective learning [4]. A systematic literature review to examine whether school health programmes improved academic success provided positive evidence for at least some programmes [5].

Similarly, another review showed positive associations between parameters of health (e.g., school-based physical activities) and academic outcomes/performance [6]. As regards the academic achievements of students, higher education institutions face dual challenges: the continuous changes in the demographic pattern of the student body; and the difficult global economic circumstances. Universities will need to match the private-sector efficiency in the provision of training and development, while simultaneously remaining as centres of learning for young people. University and college management committees are progressively more accountable for their student retention and completion rates, and other educational outcomes. An important contribution to achieving sound outcomes is a careful focus on the needs of individual learners and their satisfaction with their learning experience.

The potential for health to improve cognitive function, learning and academic achievement in children has received attention by researchers and policy makers [7]. It is widely accepted that health and well-being are essential elements for effective learning [2]. Vice versa, education is a strong predictor of lifelong health and quality of life in different populations, settings, and time [8]. However, the pathways through which education leads to better health and longer life expectancy are still not clearly understood. It is widely held, however, that education, health, and social outcomes are very closely interdependent [9]. Social and occupational status in adulthood and health status throughout life are largely determined by success in school and years of schooling [10]. Among school children, academic success, health status, and risk behaviours are cyclically interdependent. Poor school performance is associated with health-compromising behaviours and physical, mental, and emotional problems [11–13]. School performance is also compromised by poor nutrition, substance abuse, sedentary behaviour, violence, depression, and suicidality. This negative cycle, established during the school years, has large impact on success and productivity in our society [14–16].

Many factors are associated with academic outcomes [17]. Adolescents who use alcohol, tobacco or other drugs achieved lower grades, had more negative attitudes toward school, and exhibited increased absenteeism [18–21]. Furthermore, exercise seems associated with improved academic outcomes [22,23], and malnutrition additionally plays an important role in academic performance [24,25]. Hence, health surveillance questionnaires to detect students with health problems associated with academic functioning should inquire about social support, general health, physical and psychological health, study-related issues, help-seeking behaviour and life events in the past [26].

However, as regards the triad of educational satisfaction, health parameters and educational achievement, the literature suggests several gaps. First, these three important and related aspects have rarely been simultaneously examined in conjunction with each other. Most studies of health-promoting profiles of students, such as the European Health and Behaviour Survey conducted in 20 countries, did not explore the associations between health and academic achievement [27]. Indeed Al-Kandari and Vidal [28] noted that no study has yet been published on e.g., nursing students’ health promoting lifestyle profiles, particularly examining their relationship with academic performance and nursing courses.

Conversely, most studies of the factors associated with students’ educational attainment were mainly concerned with educational and/or demographic variables and did not concurrently explore the students’ health-related parameters [29]. For instance, Ofori [30] researched two variables (age, entry qualifications) on university students’ performance (educational achievement) but no health parameters were examined. Equally, surveys of students’ satisfaction with their educational experiences did not simultaneously investigate the students’ health parameters and/or educational achievement [31,32]. Likewise, studies of the impact of health and wellbeing on academic achievement and cognitive performance usually do not include any indicators of satisfaction with the educational experience [7]. Narrow examinations of the issues omit the inclusion of many important variables, jeopardising a study findings’ validity and generalisability.

Second, geographically, the majority of research that assessed the associations between health/health programmes and academic achievement was undertaken in the USA [33–35], with fewer studies from the UK or elsewhere [28]. Third, we are not aware of research that included the actual satisfaction with the educational experience as an independent variable in predicting the students’ achieved grades. When such or parallel measures of satisfaction were employed, they were used as an *outcome* rather than a predictor: Felner *et al.* [36] included student self-reported positive experience of the school climate as an outcome (achievement measure).

Fourth, the associations between health/health programmes and academic attainment were mostly examined in elementary, middle or high school children [7,36–39], rather than university/college students [28]. Fifth, in relation to the outcomes of academic achievement, many studies employed objective measure/s, usually the student’s final marks, to gauge academic performance, e.g., arithmetic scores [40]; academic grades [41]; math grades [42]; reading scores [43]; science grades [44]; or grade point average of a course of study[28]. Less studies employed, in addition to objective measures of student achievement, other *subjective* measures (e.g., the personal importance to students in achieving good grades in their studies, or the student’s subjective appraisal of their overall academic performance in comparison with their peers) in order to gauge students’ subjective academic performance and understand their subjective perceptions of their academic attainment. Finally, despite the associations between health/wellbeing and academic achievements, the literature reveals few conceptual models that could help advance the understanding of such relationships. Hence there is ample support for the critical need to study this area, especially as today’s students are the future professionals and are expected to serve as role models.

In order to address these gaps, the study detailed in this paper examined the triad of associations that are important for university students: (1) three demographic variables (age, gender, student’s financial situation); (2) a range of students’ health and health behaviour variables; (3) students’ satisfaction with their educational experience (18 different satisfaction variables); and, (4) three (two subjective and one objective) indicators of educational achievement. To the best of our knowledge, this study could be the first to simultaneously operationalise the three related notions (health, educational satisfaction, academic performance) and control for such a number of variables. For the purpose of this paper, we use the terms student ‘achievement’ and ‘performance’ interchangeably.

### 1.2. Conceptual Framework

Educational achievement can be conceptualized and measured in different ways: (1) as students’ *internal reflection* on their academic achievement in terms of the importance they attach to achieving good grades in their studies; (2) as students’ *subjective comparative* appraisal of their overall academic performance in comparison with their peers; and, (3) as an *external objective* teacher evaluation of the students’ overall academic performance as denoted by the final module marks or grades that students achieve in their course assessment/s. We employed the three measures of educational achievement (*internal reflection, subjective comparative, external objective*). Such a tri-furcated conceptualization of the varieties of educational outcomes is supported by research in the achievement goal tradition. Two types of goals have been suggested: performance goals (ego involvement or ability goals), which focus on the demonstration of competence relative to others; and mastery goals (task-involvement or learning goals), which focus on the development of competence and task mastery [45]. As such, achievement goals are conceptualized as the purpose [46] or cognitive-dynamic focus [47] of task engagement.

**Figure 1.**
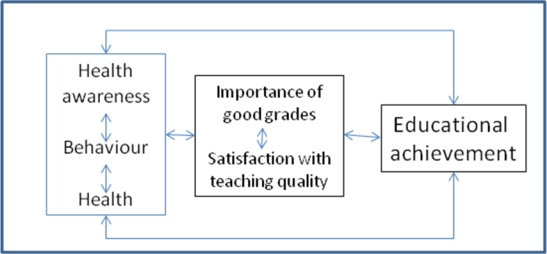
Model of reciprocal relationships between health, health behaviour and educational achievement*. *Socio-economic factors serve as background factors with potential influence on all other factors in the model.

In addition, we argue that educational achievement is largely dependent on motivational factors: students who rate the importance of good grades high would be more likely to achieve good grades; and conversely, indicators of positive performance increase the likelihood of rating the achievement of good grades as important. The satisfaction of students with their educational experience could also affect academic outcomes. Our analysis is driven by the theoretical assumption of reciprocal relationships between health awareness, health behaviours and health, motivation for academic performance and academic outcomes as depicted in Figure 1. Socio-economic factors serve in this model as background variables that may influence academic outcomes directly or through influencing, health, health behaviours or motivation. We are aware, however, that the analysis presented in this article is not able to test the relationships between the factors presented in Figure 1.

### 1.3. Aim of the Study

This study assessed the association between health awareness, health behaviour and subjective health status and three educational achievement outcomes. It was hypothesised that indicators of positive health (high health awareness, low level of complaints, high level of subjective health and health conducive behaviours) would be associated with three positive academic outcomes (*internal*: the importance of good grades; *comparative*: performance relative to other students; *external*: actual achieved module mark/grade). This research seeks to contribute to the evidence base on the importance of health and health promotion for academic outcomes and therewith for academic institutions.

## 2. Methods

### 2.1. Data Collection Procedures

Two cross-sectional studies were simultaneously undertaken among students from the University of Gloucestershire, UK during 2008–2009. Ethical approval was obtained from the university ethics committee after their review of the study design, tool, other research material, and participant information sheet which included a letter of invitation that highlighted that participation is voluntary. Both surveys employed a ‘universal sampling’ technique where all students were invited to participate.

The first study was a general student health survey [48]. The second study was a survey of students’ satisfaction with different aspects of their learning and teaching experience. The responses to the questionnaires of both studies were subsequently linked together by using the student’s registration number which participants provided when completing the questionnaires. The student’s registration number was also used to link the questionnaires to the university database and import the student’s grades. Module teachers were provided with information about the survey (aims, objectives, and the voluntary nature of participation), and were subsequently approached for permission to collect data from the students attending their module. Data protection and confidentiality were observed, and students’ grades were imported from the university database. Both questionnaires were administered towards the end of two different teaching sessions (usually the last 5 minutes in the satisfaction survey and 30 minutes in the health survey). A participant information sheet was attached to each questionnaire, and students who remained in the class to participate were asked to read the information sheet and, if they wished to participate, to remove and keep it for future reference. In this manner it was ensured that the students who stayed in the class in order to complete the survey consented to their participation and were not unwittingly coerced into the survey. The response rates were ≈80% for the health survey and the student satisfaction survey. For the analysis, we used data from students who completed both surveys (380 students).

### 2.2. Students’ Satisfaction Questionnaire and Marks

A one-page questionnaire was employed to inquire about multiple aspects of students’ learning and teaching experience. It was adopted from a tool that was developed and validated by researchers in a British University [49]. The 27 questionnaire items were closed ended items scored on 5-point scales (1 = ‘Positive Perception’ and 5 = ‘Negative Perception’). The questionnaire is detailed elsewhere [50,51] (copies of the questionnaire are available from the first author). Similar to other studies [32], students’ registration numbers linked the questionnaires to the university database, and students’ module marks were imported as percentages [31]. The satisfaction items inquired about the module team, assessment method/s, the integration of the various parts of the module, as well as the seminars, and the intellectual stimulation of the educational experience (Box 1).

Box 1. Eighteen satisfaction items assessed for each module*

1. Module ran smoothly
2. Module increased my interest in the subject
3. Module team provided opportunity to ask questions
4. Module material was well presented
5. Module was thought provoking
6. Module assessment methods were appropriate
7. Module team displayed good knowledge
8. Module team correctly assumed level of skills I had
9. Module information available at beginning of module
10. Received helpful feedback
11. Seminar group sizes were small enough
12. References needed for module available in library
13. Work required for module was appropriate
14. Module elements integrated into meaningful whole
15. Module was intellectually stimulating
16. Module is expected to be of direct use in my career
17. Module made me look at my profession differently
18. Module team styles were clear/informative/stimulating

*Each item was rated on 5-point Likert scale (1 = very positive evaluation, 5 = very negative evaluation).

### 2.3. Health & Wellbeing Questionnaire

The health & wellbeing questionnaire included socio-demographic information (e.g., gender, age, and financial situation), self-reported health data, as well as questions related to health behaviours (nutrition, physical activity, smoking and alcohol consumption), social support, quality of life and university study related questions. These items were adopted from published questionnaires [52–56]. Below, we detail only those variables that were significant when we used bivariate linear regression to screen all health related and socio-demographic variables of the questionnaire for statistically significant associations with module mark (grade) as educational outcome.

*Income sufficiency* (1 item): measured using the question adapted from Stock *et al.* [57] “Would you say the amount of money you have is…”, with a four-point response scale (1 = ‘always insufficient’, 4 = ‘always sufficient’).

*Frequency of binge drinking* (1 item): measured using the question—“Over the LAST 30 DAYS: How many times (if any) have you had five or more drinks in a row? (A “drink” is a glass of wine (ca 15 cl), a bottle or can of beer (ca 50 cl), a shot glass of spirits (ca 5 cl) or a mixed drink, with answer options ‘none’, ‘1’, ‘2’, ‘3−5’, ‘6−9’, or ‘10 or more’ times [58].

*Sleep disorder/insomnia* (1 item out of a battery of 8 health complaints): Respondents rated the question: “How often have you had sleep disorder/insomnia during the past 12 months?” on a four-point response scale (1 = ‘never’; 4 = ‘very often’), adopted from the symptoms checklist of the German Youth Health Survey [59].

*General health* (1 item): students gauged their current general health using the question: “How would you describe your general health?” with a five-point response scale (1 = ‘poor’, 5 = ‘excellent’) (adopted from [60]).

*Health awareness* (1 item): A related item asked students about their general awareness of their health [56]: “To what extent do you keep an eye on your health?”, with a four-point response scale (1 = ‘not at all’, 4 = ‘very much’).

*Educational achievement* (2 items): the health questionnaire contained two items on students’ educational achievement: “How important is it for you to have good grades at university?” (four response categories, from 1 = ‘not at all important’ to 4 = ‘very important’); and “How do you rate your performance in comparison with your fellow students?” (five response categories, from 1 = ‘much worse’ to 5 = ‘much better’).

### 2.4. Statistical Analysis

Data analysis was undertaken employing the software Statistical Package (SPSS). The datasets from both surveys were merged using student number as identifying variable.

*Principal component analysis*: in order to make the data of the student satisfaction questionnaire more manageable, principal component analysis (PCA, Varimax rotation) [61] was undertaken on the satisfaction questionnaire items to determine the number and composition of underlying dimensions to be used in subsequent analyses, by merging the original 27 questionnaire items into meaningful constructs. The PCA resulted in a four factor solution: Factor 1 (the overall quality of the teaching and the module content—9 items); Factor 2 (practical aspects and work load of the module—6 items); Factor 3 (previous interest in the subject matter and general understanding—2 items); and, Factor 4 (lecture time and module importance—2 items). We developed scores for each Factor by adding its item values and dividing it by number of items. Cronbach’s alphas for the subscales of the student satisfaction questionnaire were 0.89 (Factor 1), 0.73 (Factor 2), 0.56 (Factor 3), and 0.27 (Factor 4).

*Bivariate linear regression analysis*: was used to screen all health/wellbeing-related items and socio-demographic variables of the health questionnaire and all four Factors’ scores of the students’ satisfaction questionnaire for statistically significant associations with module mark (grade) as the educational outcome. Only those items that were significantly associated with module marks (*p*-value < 0.05) were further analysed for their association with educational achievement in the multivariate models (described below). These six items included age, income sufficiency, general health, health awareness, sleep disorder/insomnia, and frequency of binge drinking. Only Factor 1 score of the students’ satisfaction factors described above displayed a significant association with module mark (grade), explaining 1.3% of the variance in module marks, while the other three Factors each explained less than 1% of the variance. Therefore only Factor 1 was maintained for subsequent analysis. The response categories to the six items remaining in the further analyses are depicted in Table 1.

*Chi-square statistics* (χ^2^) and *t-test statistics*: were used for categorical and continuous variables respectively as we compared all the variables in Table 1 for gender differences in order to decide whether stratification for gender would be relevant in subsequent analysis.

*Multivariate linear regression analyses*: with enter mode examined the associations between importance (of good grades), performance (relative to other students), and module marks (actual achieved module grade) as the three dependent variables of educational achievement on the one hand and the selected health, demographic and educational satisfaction variables as independent variables on the other hand. As the dependent variables did not differ between males and females (Table 1), we did not stratify the analyses for gender. As described above, the selection of variables to be included in the regression models was based on significance in bivariate analysis performed with all health and demographic variables from the health questionnaire and the four Factors that emerged from the principal component analysis of the students’ satisfaction questionnaire. The independent variables included in the final models comprised students’ educational satisfaction (Factor 1), age, sleep disorder/insomnia during the last 12 months, had five or more drinks in a row last month, income sufficiency, general health, and extent of keeping an eye on one’s health. In addition, each of the three models contained the two dependent variables in the other two models as independent variables. Collinearity statistics for all three models displayed tolerance levels <1.0 and VIF values <1.3 indicating no substantial problems with collinearity between the independent variables [62]. Autocorrelation of residuals was in acceptable ranges indicated by Durbin−Watson statistics showing values of 1.86, 1.92 and 2.06 in the three models respectively.

## 3. Results

The sample comprised of 380 questionnaires (195 male and 185 female students) (Table 1).

*Educational satisfaction*: females were marginally more satisfied with their educational experience, although both genders did not significantly differ on their extent of satisfaction as measured by Factor 1 (overall quality of teaching and module content) score, reporting high to medium satisfaction (scores between 2.1 and 2.3 on a 5 point scale where 1 = high satisfaction).

*Socio-demographic variables*: females were on average ≈3 years older than their male counterparts. Financially, slightly more than one third of the sample felt that their income was mostly or always insufficient. About 20% of the sample did not binge alcohol in the month preceding the health survey. Significantly more women than men reported that they had not had five or more drinks in a row in the last month on six or more occasions.

*General health, health awareness and health complaints:* about one fifth of the sample sometimes or very often experienced a sleep disorder or insomnia during the year preceding the surveys. There were however, significant differences in general health, where females were about twice more likely to report excellent health whilst males were about twice more likely to feel that their health was fair. This was paralleled by slightly more females who reported that they kept a constant eye on their health.

*Educational outcomes*: most of the sample felt that it was either very or somewhat important to have good grades at University, and about 60% of the students rated their academic performance as being the same when compared with their peers and fellow students. The actual achieved grade (module marks) were slightly above 50% and did not differ between the genders.

*Are students’ educational satisfaction and health parameters associated with the three educational outcomes?*: multivariate linear regression (Table 2) suggested that the variables under study exhibited selective associations with each of the three educational outcomes (importance of good grades; student’s performance relative to peers; and student’s module mark/ actual achieved grade). Firstly, student’s satisfaction with the learning and teaching experience was not associated with any of the three educational outcomes. As regards demography, with increasing age, students attached less importance to accomplishing good grades in their studies, but actually achieved better grades in their assessments. Financially, students with sufficient income attached less importance to attain good grades, although they felt that their performance was better relative to peers. Income, however, had no effects on the actual grade that was achieved. In connection with health behaviours/lifestyle features, alcohol binging was negatively associated with the three educational outcomes, although it was significant only in relation to the importance of achieving good grades. As regards health complaints, sleep disorder or insomnia during the year preceding the survey was negatively associated with the three educational outcomes, but its effect was only close to significance as regards the student’s module mark (actual achieved grade). When it came to the health status variables, students feeling better general health were more likely to rate their academic performance as being better relative to their fellow peers. Similarly students with high health awareness (those who kept a constant eye on their health) felt that it was important for them to attain good grades on the courses they were attending. Finally each of the three educational outcomes under study (importance of good grades; student’s performance relative to peers; and student’s module mark/ actual achieved grade) was significantly associated with the other two educational outcomes, with the exception of the relationship between performance relative to peers and module mark which was not significant.

## 4. Discussion

We examined the triad of associations that are important for university students: satisfaction with their educational experience; their demographic variables; their health/wellbeing and health behaviours; and, three indicators of their educational achievement. Among school children, academic success, health status, and risk behaviours are cyclically interdependent. Given that investigating a few isolated variables obscures confounding relationships [63], this study links these various features together in order to capture the wider landscape of university students’ wellbeing, educational satisfaction and achievement.

As regards satisfaction, students exhibited high to medium satisfaction with their educational experiences. This is in agreement with other surveys where, depending on the aspect of the learning experience that was inquired about, between 26 and 97% of students reported that they were satisfied with their educational experience [32]. There were also no gender differences in the reported satisfaction levels, in support of both El Ansari & Oskrochi [32] who found no gender differences in a study of one university in the UK, and Kerridge & Mathews [49] who reported gender differences in only one out of 12 satisfaction questions that they inquired about in the UK.

The substantial pressure on children, parents, and schools to maximize academic performance [64] has resulted in a growing interest in the potential factors that impact on educational outcomes. About 20% of the sample experienced (sometimes or very often) a sleep disorder or insomnia during the year preceding the survey. Health complaints limit students’ capacity to perform adequately at university [26,65]. Our findings support such assertions, as sleep disorder/insomnia was significantly and negatively associated with the students’ module mark (actual achieved grade). However, the repercussions of disappointing academic results may be manifold, as such disappointments might also further affect existing health problems, initiating a vicious circle in which health conditions and study problems negatively influence each other [26]. This highlights the importance of the early detection of such health complaints through university health programmes.

Although the majority of students in our sample perceived their health as good to excellent, and kept (to some extent or very much) an eye on their health, perceived health and health awareness were associated either with students’ importance of good grades or how they rated their own academic performance. A close link between health, well-being and academic performance has also been acknowledged by others [2], suggesting the need for quality programmes at universities which take into account health as an important factor.

Other studies have found that poor school performance was associated with health-compromising behaviours and physical, mental, and emotional problems [11–13]. Our study findings supported such a link, where the frequency of binge drinking in the last month was negatively correlated with the importance of good grades. However, it is also important to note that in our sample, other health behaviours such as smoking, physical activity or nutritional factors did not display, in the bivariate analyses, significant associations with module mark as educational outcome and were therefore excluded from further analyses. Others have highlighted the relevance of physical activity and healthy nutrition for good school performance in children [22,24,25]. Nevertheless, the lack of significant associations between physical activity and module mark are conceivable. For instance, although many studies have shown positive relationships between academic achievement and physical activity [66,67], few have shown no correlation [68] or an inverse relationship [69].

The point to note is that, in university students, the association between binge drinking and academic achievement seems to be the most relevant relationship. From the present cross-sectional study, no conclusions can be made in terms of causal relationships, but it is very likely that the relationship between alcohol use and educational achievement is reciprocal as our conceptual model suggests. For instance, taking the relationship between physical activity and school achievement in children as a parallel example, increased activity levels might be related to increased self-esteem, which could be expected to improve classroom behaviour as well as academic performance [67]. It might be that alcohol binge drinking might operate in the opposite direction. Moreover, recent reports found the first direct evidence that short-term binge drinking can generate evident cerebral dysfunction undetectable by behavioural measures alone. The observed latency abnormalities, similar to those observed in long-term alcoholism, comprise an electrophysiological marker of slowed cerebral activity associated with binge drinking [70]. Moreover, the importance of the college context has been shown, where heavy drinking does in fact predict student attrition [71]. Indeed in a nationally representative sample of college and university students in the USA, alcohol use was rated as one of the top 10 hindrances to students’ academic performance [72]. Problematic drinking during college has been identified to have an effect on several individual-level outcomes e.g., alcohol use disorder in students may result in visuospatial deficits [73].

An interesting finding was related to the multiple indicators of student achievement that the present study mobilized. Table 2 showed that many of the health/ wellbeing, demographic and satisfaction variables we explored were associated with particular achievement indicator/s but not with the others. This suggested that caution needs to be exercised when researchers and practitioners select a given outcome measure of student achievement, and where possible, it could be beneficial to employ more than one outcome of student achievement. Further, even though the accomplished grade (module mark) is seen as an objective measure, it could still be viewed as subjective as it is also dependent on the teacher/s who mark the student’s assignment and allocate a grade to the student’s coursework. Despite this, such indicators that employ the accomplished grade (module mark) of pupil/student achievement have been widely used, as an isolated marker, in order to gauge academic performance [20,28,40,42–44].

If one compares the three models of outcomes of educational achievement with each other (Table 2), it becomes compelling that the educational outcome “importance of good grades” could be best explained by the covariates used in the model (only 3 covariates did not reach statistical significance). Conversely, the outcome “performance relative to peers” showed fewer associations with the covariates (6 non-significant results); and “module mark” the least (7 non-significant results). In agreement, the amount of variance of educational achievement that is explained by the three models decreased from 21% to 9%. Although this does not verify our conceptual framework, it provides support for the framework as depicted in Figure 1, assuming that the importance of good grades is mediating the influence of health and health behaviours on educational achievement. All the three models showed significant or at least almost significant associations between the three measures of academic achievement, again indicating support to the reciprocal relationships between importance of good grades and educational achievement as suggested in our conceptual framework.

Financial restrictions place pressure on students, as many of them are forced to work besides studying. This represents a ‘double burden’ with potentially harmful effects on educational achievements. In our sample, more than 30% of students perceived his/her income as insufficient. Our findings suggested that satisfaction with income was correlated with better educational achievement in terms of performance compared to peers. Income insufficiency could be a main reason where students are obliged to study part time. For instance, many nursing students are working [74,75], usually citing financial hardship as the main motivating factor [76]. Further, for such part-time students, research has shown significant differences between full- and part-time students as regards their satisfaction with their educational experience at university [77]. Economic factors as predictors for academic outcomes, however, cannot be addressed in health programmes alone, but need to be addressed at the policy macro level and politically, in terms of scholarship programmes and/or financial support programmes for disadvantaged students, or perhaps monetary incentives for high-achieving students. Interestingly, satisfaction with income was also correlated with lower importance of good grades suggesting that very high income could have diminishing effects on students’ educational achievement. This would again support incentive systems where monetary incentives are linked to achievement.

This study has limitations, and generalization of the findings requires caution. We surveyed one university in the UK. Self-reported data could be subject to recall bias, sociability and social desirability. Despite our broadening of the data collection in an attempt that the selection of students in this study would be representative of their university, our sample remains a convenience sample. Such convenience samples are not uncommon in student surveys e.g., in Hong Kong [78], in the USA [79], or Australia [80]. Similarly, in the USA, post secondary institutions (universities and colleges) self-selected themselves to participate in the American College Health Association National College Health Assessment survey [73]. Other limitations are related to the use of empirical measures of health, the endogeneity of health outcomes and their interactions with risky behaviours, and that strong positive correlations between multiple health disorders and health behaviours that might exist in the data present a major challenge for researchers interested in identifying the individual impacts of poor health conditions on academic performance [81]. In addition, one of our outcome measures (module mark) is based on the marks in only one module and does not take into account the variations of academic performance across different modules. Further research would need to explore how different health and wellbeing variables are associated with different measures of student achievement, and the mediating variables for such relationships and should attempt to statistically test the relationships suggested in the conceptual model.

## 5. Conclusion

In conclusion, the findings from this study support a conceptual framework suggesting reciprocal relationships between health, health behaviours and educational achievement. Even when health, health awareness and health behaviour variables were not directly linked to module marks, they were associated with some of the determinants of module marks such as the importance of good grades. The results support a focus on comprehensive health programmes at universities that take into account the multiple factors influencing students’ achievement. Comprehensive health promotion programmes may have the potential to influence relevant predictors of educational achievement in university students and therewith do not only add to population health, but contribute to the core business of higher education institutions.

## Figures and Tables

**Table 1.** Demographic and other selected characteristics of the study sample†.

**Variable**	**Whole sample** (N = 380)	**Male** (N = 195)	**Female** (N = 185)	***p*** value
**Satisfaction Questionnaire**				
**Educational Satisfaction‡**	2.19 (0.72)	2.27 (0.75)	2.17 (0.74)	NS
**Health & Wellbeing Questionnaire**				
**1. Socio-demographic Variables**				
**Age in years: Mean (SD)**	22.86 (7.5)	21.22 (4.7)	24.58 (9.4)	<0.001
**Financial: Income Sufficiency**				NS
Always sufficient	9.6	10.5	8.6	
Mostly sufficient	52.9	55.5	50.0	
Mostly insufficient	25.8	23.0	28.7	
Always insufficient	11.8	11.0	12.6	
**2. Health & Wellbeing Variables**				
**Alcohol Consumption: Had five or more drinks in a row last month**				<0.001
None	20.4	13.0	28.2	
1 time	12.9	8.9	17.1	
2 times	12.9	14.1	11.6	
3−5 times	26,5	27.1	26.0	
6−9 times	15.8	19.8	11.6	
10 or more times	11.5	17.2	5.5	
**Health Complaints: Sleep disorder/ Insomnia during the last 12 months**				NS
Never	52.4	58.5	45.9	
Rarely	25.3	22.6	28.1	
Sometimes	16.6	14.4	18.9	
Very often	5.8	4.6	7.0	
**General Health**				0.002
Excellent	10.3	14.4	5.9	
Very good	39.5	43.6	35.1	
Good	44.5	39.0	50.3	
Fair	4.7	2.1	7.6	
Poor	1.1	1.0	1.1	
**Health Awareness: Extent of keeping an eye on one’s health**				0.012
Not at all	0.8	1.0	0.5	
Not much	10.3	13.8	6.6	
To some extent	61.4	53.8	69.4	
Very much	27.5	31.3	23.5	
**3. Educational Outcome Variables**				
**Importance of having good grades at University**				NS
Very important	64.1	61.7	66.7	
Somewhat important	34.3	36.3	32.2	
Not very important	1.3	1.6	1.1	
Not at all important	0.3	0.5	0.0	
**Rating of one’s academic performance in comparison with fellow students**				NS
Much better	2.1	3.1	1.1	
Better	21.3	22.3	20.2	
The same	62.5	62.2	62.8	
Worse	13.8	12.4	15.3	
Much worse	0.3	0	0.5	
**University Computerised Student Records**				
**Module Mark- actual achieved % grade: Mean (SD)**	54.56 (12.9)	53.90 (12.1)	55.68 (12.4)	NS

† *p*-value based on chi-square statistics for categorical variables and t-test statistics for continuous variables;

‡ cells depict Factor 1 score and (standard deviation); NS: Not significant.

**Table 2.** Regression Models For Three Different Indicators of Educational Achievement†.

**Variable**	**Importance (of good grades)**		**Performance (relative to peers)**		**Module Mark (Actual achieved grade)**	
	**Standardized β**	***p* Value**	**Standardized β**	***p* Value**	**Standardized β**	***p* Value**
Higher educational satisfaction (Factor 1)‡	−0.096	NS	0.079	NS	−0.055	NS
Older age	−0.234	<0.001	0.022	NS	0.204	<0.001
Higher level of income sufficiency	−0.179	<0.001	0.139	0.009	0.095	NS
Higher frequency of five or more drinks in a row	−0.500	0.004	−0.009	NS	−0.034	NS
Higher frequency of sleep disorder/ insomnia	−0.008	NS	−0.003	NS	−0.109	NS
Higher level of general Health	−0.012	NS	0.204	<0.001	−0.037	NS
Higher extent of keeping an eye on one’s health	0.150	0.004	0.038	NS	0.063	NS
Higher importance of having good grades	—	—	0.289	<0.001	0.129	0.033
Higher rating of own academic performance	0.278	<0.001	—	—	0.114	NS
Higher achieved module mark	0.112	0.033	0.103	NS	—	—
**Adjusted R^2^ of the model**	**0.21**		**0.18**		**0.09**	

† Importance: importance of having good grades at University; Performance: rating of one’s academic performance in comparison with fellow students; Module Mark: actual achieved module grade in %;

‡ Satisfaction with the module’s learning and teaching experience; NS: Not significant.
